# Atraumatic Idiopathic Compartment Syndrome Requiring Emergent Fasciotomy: A Case Report and Literature Review

**DOI:** 10.7759/cureus.57208

**Published:** 2024-03-29

**Authors:** Danielle Markus, Andrew S Bi, William Neal, Benjamin Fiedler, Nirmal Tejwani

**Affiliations:** 1 Orthopedic Surgery, New York University (NYU) Langone Orthopedic Hospital, New York, USA; 2 Orthopedics, New York University (NYU) Langone Orthopedic Hospital, New York, USA

**Keywords:** hematoma, atraumatic, emergency fasciotomy, emergent fasciotomy, compartment syndrome leg

## Abstract

A 42-year-old male with no past medical history presented to an emergency room with increasing pain and swelling of his left lower extremity over 48 hours with no preceding trauma. A computed tomography scan demonstrated a hematoma (20 cm × 4 cm × 10 cm) present within the gastrocnemius-soleus complex. Acute compartment syndrome (ACS) was diagnosed clinically, confirmed intraoperatively with an arterial line transducer, and treated with emergent fasciotomy. Extensive workup found no evidence of coagulopathy or source of bleeding. This case presents a patient with ACS secondary to an atraumatic gastrocnemius hematoma discovered in the emergency room with no history of coagulopathies or anticoagulation.

## Introduction

Acute compartment syndrome (ACS) occurs when there is increased pressure within a closed osteofascial compartment resulting in reduced local circulation, usually occurring after trauma, such as fractures or crush injuries, or iatrogenic reasons, such as peripheral line extravasation, or reperfusion injury [1]. It is a relatively rare condition with an incidence of 7.3 per 100,000 males and 0.7 per 100,000 females [2]. ACS is commonly defined as a delta pressure of less than or equal to 30 mmHg relative to the patient’s diastolic blood pressure; however, diagnosis is based largely on clinical examination. Positive diagnosis is considered a surgical emergency requiring fasciotomy, as failure to restore circulation within 4-12 hours can lead to soft tissue necrosis and permanent disability [3]. As a result, early identification of ACS is crucial and requires a high index of suspicion based on risk factors, pathophysiology, and clinical manifestations.

Atraumatic compartment syndrome is uncommon and rarely reported in the literature [4]. This case report describes an otherwise healthy male with no past medical history who presented with atraumatic compartment syndrome of the superficial posterior compartment of the leg secondary to an expanding hematoma requiring emergent fasciotomy. The patient was informed that data concerning the case would be submitted for publication and provided informed consent.

## Case presentation

A 42-year-old male with no past medical history and taking no medications presented to the emergency department with complaints of pain and swelling in his left leg over the last 48 hours. The patient noticed the pain while walking to work the day prior, but denied any trauma or injury to the leg. The pain did not resolve with rest. He stated the pain and swelling were progressively worsening, and he was now having difficulty ambulating, prompting him to come to the emergency department. On initial emergency department evaluation, the patient’s left leg was moderately edematous compared to the contralateral side. Compartments were firm but compressible, and he was tender to palpation over the posterior leg. There was no pain with passive stretching of the great toe, but the patient endorsed pain with passive dorsiflexion of the ankle. The patient was neurovascularly intact distally, and a complete secondary examination was negative for other orthopedic injuries.

**Figure 1:**
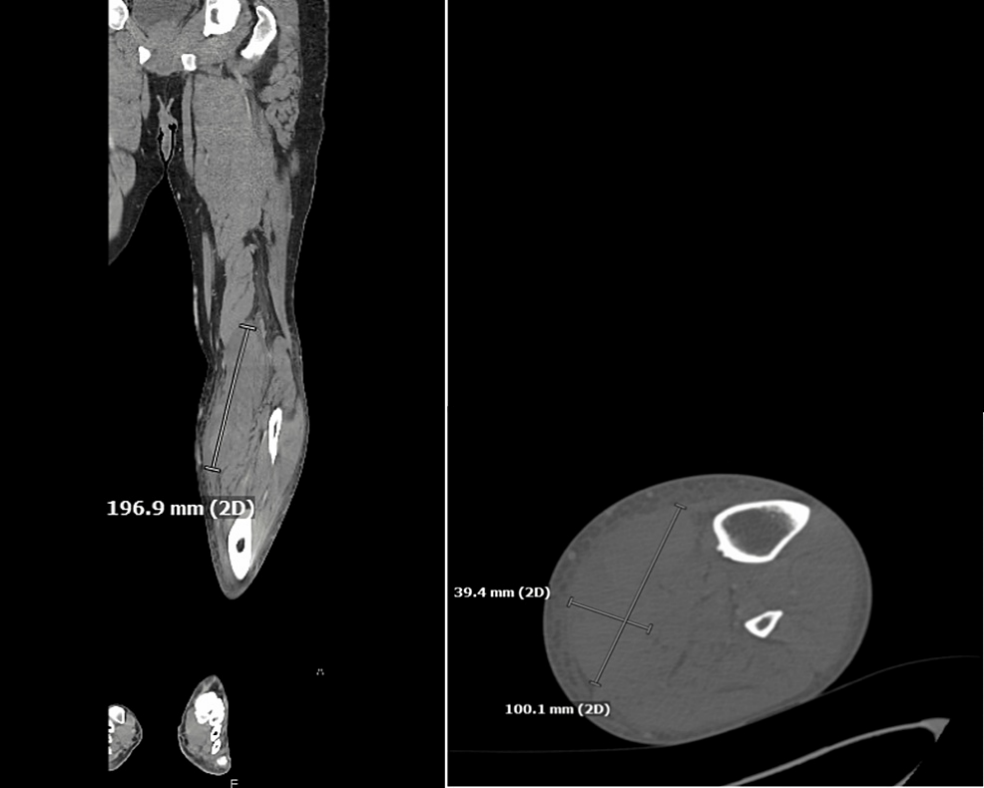
Coronal and axial CT of the left lower extremity with intravenous contrast demonstrates a hematoma measuring 100.1 mm anterior-posteriorly × 39.4 mm transversely × 196.9 mm cranio-caudally.

Further workup included Doppler, X-ray, and a CT scan of the left lower extremity (Figure 1). Doppler ultrasound showed no evidence of a deep vein thrombosis. X-rays demonstrated soft tissue swelling with no acute fractures or dislocations, and CT demonstrated a crescentic hyperdense collection along the medial gastrocnemius muscle in the left leg consistent with a hematoma measuring 10 cm anterior-posteriorly × 4 cm transversely × 20 cm craniocaudally. While the examination was abnormal, there was no concern for ACS at that time, and the patient was admitted to observation with compartment checks.

The patient’s clinical picture remained stable over the following 12 hours, and he denied any worsening pain or swelling. Sixteen hours after the initial consult, the patient was noted to have an interval increase in swelling and tension of the skin over the posterior leg when compared to the examination done four hours prior. He was now endorsing paresthesias in the distribution of the tibial nerve and worsening pain with passive dorsiflexion and plantarflexion of the ankle. He was otherwise neurovascularly intact distally. Given the worsening clinical picture, the patient was taken to the operating room for emergent fasciotomy.

**Figure 2:**
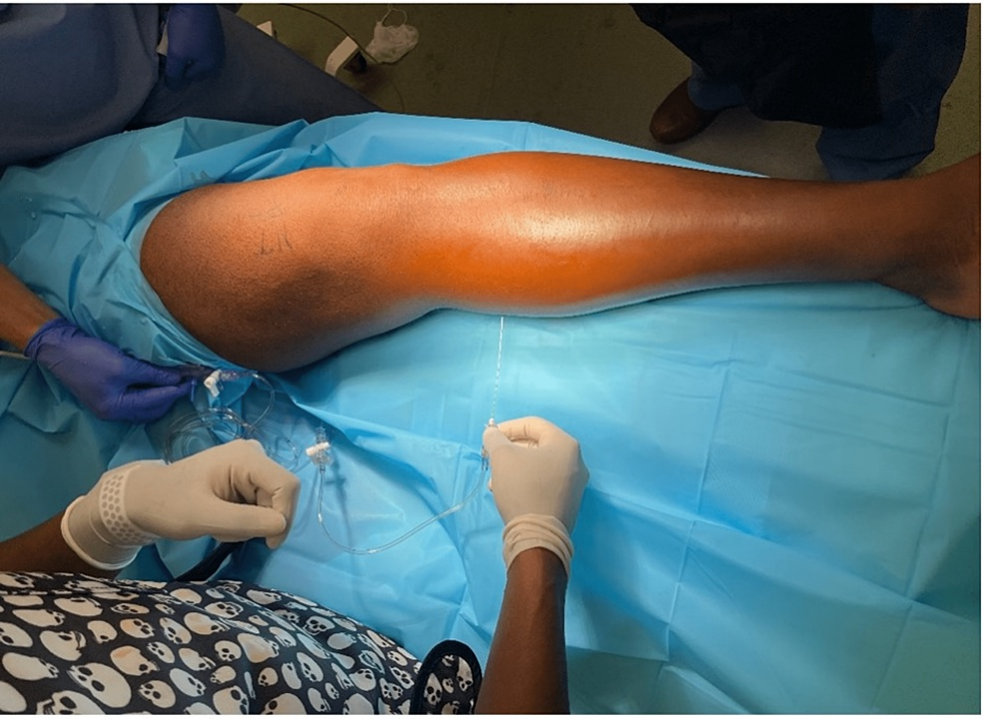
Intraoperative compartment pressure check of the superficial posterior compartment.

Compartment pressure testing was performed in the operating room using the arterial line transducer technique (Figure 2) [5]. Pressures were noted to be elevated primarily in the superficial posterior compartment with a delta pressure of -5 mmHg relative to diastolic blood pressure (diastolic blood pressure 81, absolute compartment pressure 86 mmHg), and the deep posterior compartment with a delta pressure of 10 mmHg (absolute compartment pressure 71 mmHg). Anterior and lateral compartments were measured to be delta 50 mmHg.

**Figure 3:**
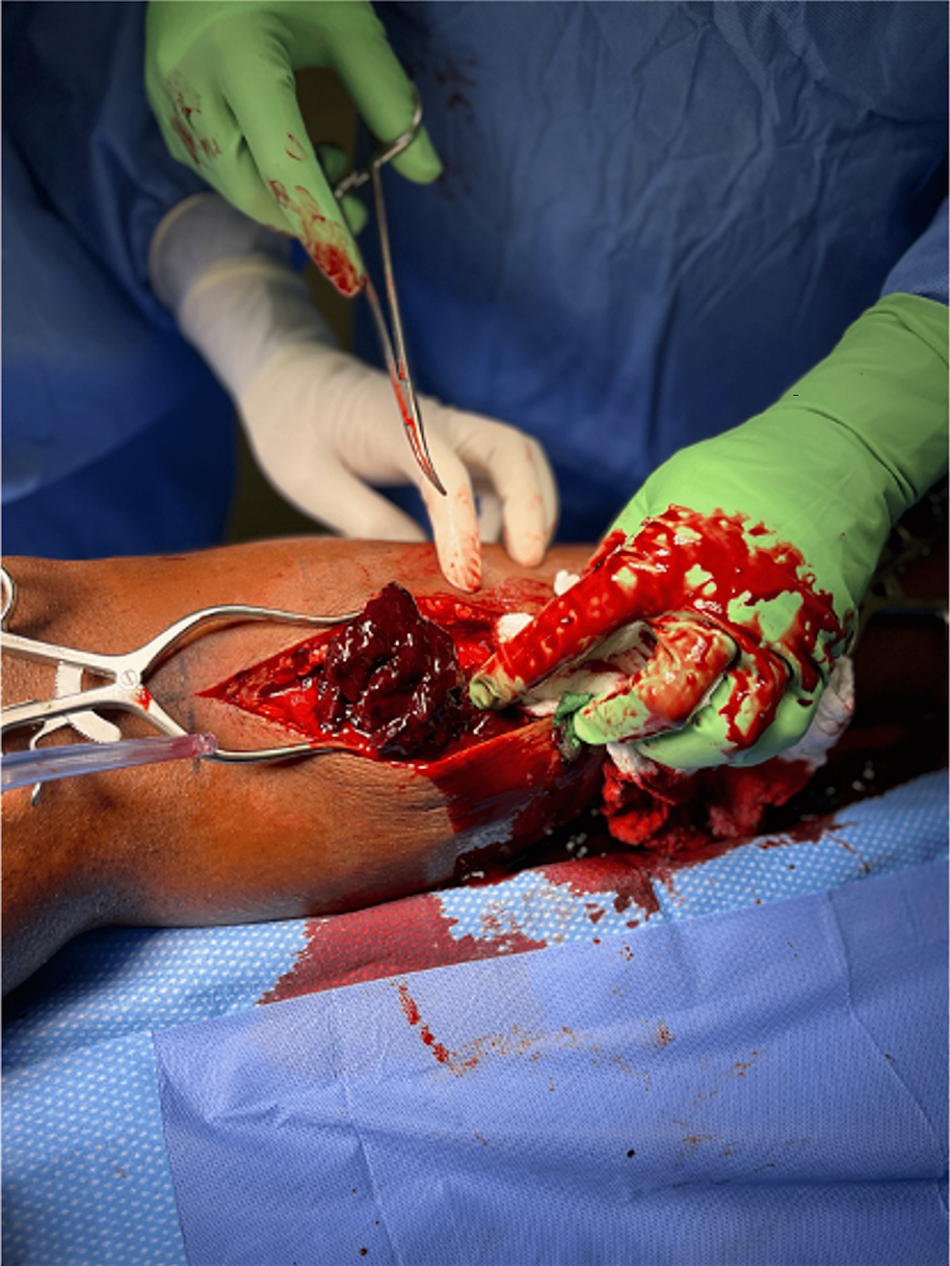
Image from the operating room demonstrating the initial clot evacuated from the superficial posterior compartment.

**Figure 4:**
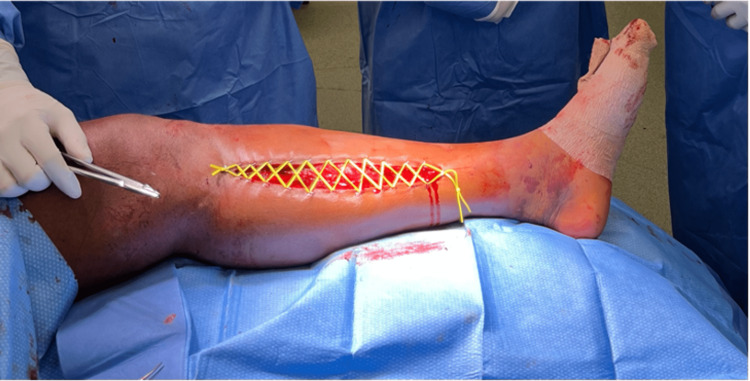
Demonstration of medial closure.

Fasciotomies of all four compartments were immediately performed using a two-incision approach starting with a 20 cm incision along the posterior aspect of the medial tibia. Approximately 300 cc of hematoma was evacuated from the superficial compartment (Figure 3), with minimal amounts of necrosis noted superficially on the medial head of the gastrocnemius. The remainder of the gastrocnemius, as well as the posterior deep compartment after the incision of the soleal bridge, appeared healthy and contractile, with no apparent source of arterial or obvious venous bleeding noted. After the superficial and deep posterior compartment release, the anterior and lateral compartments were noted to be soft and compressible. An incision measuring about 8 cm in length was taken over the anterolateral compartment through the skin, subcutaneous tissue, and fascia. The fascia of both the anterior and lateral compartments were released in a prophylactic manner. Muscles in these compartments were noted to be viable and contractile. The lateral incision was closed. The medial incision was approximated using the Roman sandal vessel loop technique (Figure 4). A vacuum-assisted closure device was then applied for dressings.

The hematology service was consulted to determine if a coagulopathy or underlying disorder could have contributed; however, the resulting workup was negative. His HbA1c was also normal. The vascular surgery team performed a CT angiogram with arterial and venous phases without an apparent source of the bleeding. Four days later, the patient was taken back to the operating room for irrigation and debridement with definitive closure.

**Figure 5:**
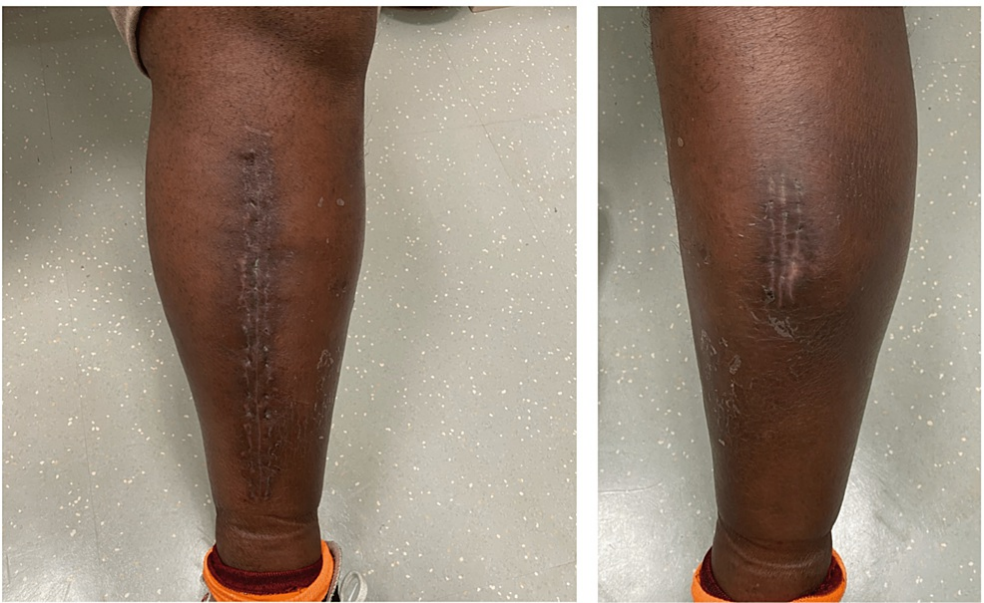
At the one-month follow-up showing medial (left) and lateral (right) incisions.

At the one-month follow-up, the patient had no residual symptoms and the incision had healed appropriately (Figure 5).

## Discussion

Compartment syndrome is a surgical emergency as delay in treatment can result in an increased likelihood of adverse outcomes such as higher rates of morbidity and mortality. While trauma is the most common cause of ACS with an associated fracture in 69% of patients [6], a variety of atraumatic etiologies have been identified in the literature, including prolonged immobilization secondary to substance abuse, loss of consciousness, or operative procedure [7-9]; long-standing uncontrolled diabetes [10]; use of anticoagulation medication [11]; or an undiagnosed bleeding disorder [12]. The average time from symptom onset to diagnosis of ACS in patients without a fracture is approximately two days, similar to what was seen in the current presentation [13]. The wide variance in patient population and injury patterns complicates diagnostic algorithms, as the clinician must maintain a high degree of suspicion even in those without a fracture or high-energy mechanism [14]. When compartment syndrome is due to atraumatic causes, clinicians often have a lower suspicion of index, and therefore, patients may be subject to longer times before receiving fasciotomy [15].

Calf strains are a relatively common injury in athletes, typically presenting with the acute onset of focal calf pain and ecchymosis after injury [14]. While typically treated conservatively, there have been a small number of cases published in the literature regarding a traumatic gastrocnemius rupture leading to ACS in athletes. However, even more rarely, cases have been reported related to a hematoma formation in the setting of atraumatic rupture of gastrocnemius or soleus muscle in nonathletes leading to ACS. Sit et al. [16] described a 55-year-old male with calf pain after chasing a bus, who was noted to have a gastrocnemius tear leading to ACS. Similarly, O’Neill et al. [14] described a 62-year-old male who was diagnosed with ACS after a short track race, in which a soleus muscle tear resulted in 400-500 cc of hematoma. Tao et al. [17] published the case of a 47-year-old male who was diagnosed with ACS secondary to a gastrocnemius tear after stepping down from a truck bed. Notably, however, is that these described patients “felt a pop” despite the atraumatic mechanism. In comparison, the current case is unusual in its lack of pertinent risk factors or a known inciting injury (a “pop”).

It is commonly taught to diagnose compartment syndrome with the 6 Ps: pain, pallor, paresthesia, paralysis, pulselessness, and poikilothermia. However, these diagnostic criteria have been called into question in a growing number of studies. Ulmer et al. [18] demonstrated that the sensitivity of these symptoms can be as low as 13-19%. However, when present, they have a specificity of up to 98%. Similarly, in the current presentation, the patient had no pain with the passive stretch of his big toe, despite having pathologically high pressure in both the superior and deep compartments.

## Conclusions

This case presents a patient with ACS secondary to an atraumatic gastrocnemius hematoma discovered in the emergency room with no history of coagulopathies or anticoagulation.

## References

[1] Via AG, Oliva F, Spoliti M, Maffulli N (2015). Acute compartment syndrome. Muscles Ligaments Tendons J.

[2] Torlincasi AM, Lopez RA, Waseem M (2022). Acute Compartment Syndrome. http://www.ncbi.nlm.nih.gov/books/NBK448124/.

[3] Sayar U, Ozer T, Mataracı I (2014). Forearm compartment syndrome caused by reperfusion injury. Case Rep Vasc Med.

[4] Cara JA, Narváez A, Bertrand ML, Guerado E (1999). Acute atraumatic compartment syndrome in the leg. Int Orthop.

[5] Wilson SC, Vrahas MS, Berson L, Paul EM (1997). A simple method to measure compartment pressures using an intravenous catheter. Orthopedics.

[6] McQueen MM, Gaston P, Court-Brown CM (2000). Acute compartment syndrome. Who is at risk?. J Bone Joint Surg Br.

[7] Parzych L, Jo J, Diwan A, Swart E (2019). "Found down" compartment syndrome: experience from the front lines of the opioid epidemic. J Bone Joint Surg Am.

[8] Adib F, Posner AD, O'Hara NN, O'Toole RV (2022). Gluteal compartment syndrome: a systematic review and meta-analysis. Injury.

[9] Markus DH, Mojica ES, Blaeser AM, Avila A, Strauss EJ (2022). Acute well-leg compartment syndrome after meniscal allograft transplantation and revision ACL reconstruction: a case report. JBJS Case Connect.

[10] Woolley SL, Smith DR (2006). Acute compartment syndrome secondary to diabetic muscle infarction: case report and literature review. Eur J Emerg Med.

[11] Chavez G, Choi J, Fogel N, Jaramillo JD, Murphy M, Spain D (2019). Atraumatic acute forearm compartment syndrome due to systemic heparin. Trauma Surg Acute Care Open.

[12] Donaldson J, Goddard N (2015). Compartment syndrome in patients with haemophilia. J Orthop.

[13] Mueller JW, Mcleod CB, Rabenhorst BM (2022). Isolated acute lateral compartment syndrome in an adolescent athlete: a case report. JBJS Case Connect.

[14] O'Neill CN, Johnsen PH, Stefanski JT, Toney CB (2021). Acute compartment syndrome after isolated soleus tear in an elderly recreational athlete. Geriatr Orthop Surg Rehabil.

[15] Lamplot JD, Wang D, Weiss LJ (2021). Lower extremity compartment syndrome in National Football League athletes. Sports Health.

[16] Sit YK, Lui TH (2015). Acute compartment syndrome after medial gastrocnemius tear. Foot Ankle Spec.

[17] Tao L, Jun H, Muliang D, Deye S, Jiangdong N (2016). Acute compartment syndrome after gastrocnemius rupture (tennis leg) in a nonathlete without trauma. J Foot Ankle Surg.

[18] Ulmer T (2002). The clinical diagnosis of compartment syndrome of the lower leg: are clinical findings predictive of the disorder?. J Orthop Trauma.

